# Dissociation in Optokinetic Stimulation Sensitivity between Omission and Substitution Reading Errors in Neglect Dyslexia

**DOI:** 10.3389/fnhum.2013.00581

**Published:** 2013-09-17

**Authors:** Roberta Daini, Andrea Albonico, Manuela Malaspina, Marialuisa Martelli, Silvia Primativo, Lisa S. Arduino

**Affiliations:** ^1^Psychology Department, University of Milano-Bicocca, Milano, Italy; ^2^Psychology Department, University of Rome La Sapienza, Rome, Italy; ^3^Neuropsychology Unit, IRCCS Fondazione Santa Lucia, Rome, Italy; ^4^Department of Human Sciences, University LUMSA & Institute of Cognitive Sciences and Technologies, CNR, Rome, Italy

**Keywords:** unilateral spatial neglect, optokinetic stimulation, neglect dyslexia, neuropsychological rehabilitation, eye movements

## Abstract

Although omission and substitution errors in neglect dyslexia (ND) patients have always been considered as different manifestations of the same acquired reading disorder, recently, we proposed a new dual mechanism model. While omissions are related to the exploratory disorder which characterizes unilateral spatial neglect (USN), substitutions are due to a perceptual integration mechanism. A consequence of this hypothesis is that specific training for omission-type ND patients would aim at restoring the oculo-motor scanning and should not improve reading in substitution-type ND. With this aim we administered an optokinetic stimulation (OKS) to two brain-damaged patients with both USN and ND, MA and EP, who showed ND mainly characterized by omissions and substitutions, respectively. MA also showed an impairment in oculo-motor behavior with a non-reading task, while EP did not. The two patients presented a dissociation with respect to their sensitivity to OKS, so that, as expected, MA was positively affected, while EP was not. Our results confirm a dissociation between the two mechanisms underlying omission and substitution reading errors in ND patients. Moreover, they suggest that such a dissociation could possibly be extended to the effectiveness of rehabilitative procedures, and that patients who mainly omit contralesional-sided letters would benefit from OKS.

## Introduction

Unilateral spatial neglect (USN) is defined as a neuropsychological disorder in which patients fail to detect or identify objects or to execute movements in the portion of space contralateral to the lesion side (Vallar, 2001; Halligan et al., 2003). USN is a syndrome that presents multiple symptoms (e.g., personal, peripersonal, and extrapersonal neglect, “motor” and “perceptual” neglect) and involves multiple cognitive functions (e.g., spatial cognition, attention, visual awareness). So, despite the fact that in the literature, particularly regarding rehabilitation, it has been treated as a unitary disorder, it is most likely due to multiple etiopathogenetic mechanisms.

Unilateral spatial neglect has a 40–80% incidence in acute stroke patients, and although evidence-based evaluation of rehabilitation of USN (e.g., Rohling et al., 2009) indicates positive effects, only a few studies have examined the effectiveness of treatments across several tasks and patients for specific domains of cognitive functioning. For example, adopting a meta-analytic approach and estimating effect sizes, Rohling et al. (2009) reported the effectiveness of cognitive rehabilitation with different treatments for focal impairments within cognitive domains. The results for the neglect syndrome show that gains are moderate in size (it persists chronically in one third of patients) and domain specific, indicating sufficient evidence for the effectiveness of visuo-spatial training in these patients. Overall, indications from the literature call for selective training on explorative symptoms (Bowen and Lincoln, 2007; Rohling et al., 2009).

Recently, Zoccolotti et al. (2011) made a systematic evidence-based review of the studies on rehabilitation training of neglect disorders up to 2007. They considered top-down techniques, such as visuo-spatial orientation training, characterized by a conscious learning of strategies to compensate for the lack of attention toward the neglected side of space, as well as bottom-up techniques, consisting of sensory stimulation aiming at “re-balancing” the representation of space. In particular, they considered prism adaptation, optokinetic stimulation (OKS), caloric vestibular stimulation, transcutaneous electrical neural stimulation, bio-feedback, eye patching, and some neuropharmacological approaches.

According to the analysis of the literature, the most highly recommended training is visuo-spatial orientation training and, among the bottom-up techniques, prism adaptation. However, the general quantitative approach used in the review (Zoccolotti et al., 2011) did not clarify which symptoms showed by the patients were really influenced by the different treatments.

As indicated in Rossetti and Rode (2002) and, more recently, in reviews about USN rehabilitation (Luauté et al., 2006; Kerkhoff and Schenk, 2012), it seems that some sensory and cognitive therapies have different impacts on different USN symptoms.

Prism adaptation seems to have a general rehabilitative effect, but no effect was found on perceptual tasks, such as single words reading (McIntosh et al., 2002), perception of chimeric faces (Ferber et al., 2003), object size estimation (Dijkerman et al., 2003), and haptic perception (Serino et al., 2007).

When different rehabilitation techniques are combined, it is possible to see dissociable effects, so that for example, different patients with both anosognosia and neglect respond differently to the combined treatments (Beschin et al., 2012).

Saevarsson et al. (2011) in their review conclude that “different therapeutic techniques used in combination that are applied repeatedly may currently be the most promising approach to treating the disorder and most likely produce the strongest and longest-lasting effects,” but they state also that “… the current state of knowledge of specific aspects of neglect and their interaction for individual patients is not sufficient to serve as a basis for selecting a particular therapy.”

While sharing the latter claim, however, we believe that it is precisely the direction in which the rehabilitation of the neglect syndrome will go in the future.

In this single cases study, we propose an approach to the rehabilitation of neglect more similar to that used with other neuropsychological disorders such as aphasia, where symptoms associated with comprehension, repetition, and production deficits, as indicators of the specific mechanisms that are compromised, are treated with specific procedures.

In particular, we focused on the acquired reading disorder often associated with USN, neglect dyslexia (ND). This symptom shows a high co-morbidity with USN and the reading impairment co-occurred with other spatial deficits in 40% of patients (Lee et al., 2009).

Neglect dyslexia determines errors in reading the contralesional side of words, sentences, and texts. Nevertheless, most experimental studies on ND are primarily concerned with single word reading where patients misread letters that occupy the contralateral side of the visually presented stimulus. The most common errors in single word reading are: (i) substitutions [e.g., the target word *albero* (tree) read as a non-word like *pobero*] and (ii) omissions [e.g., the target word *famiglia* (family) read as *miglia* (miles)]. However, for some patients a predominance of substitution errors has been reported (e.g., Ellis et al., 1987; Behrmann et al., 1990; Riddoch et al., 1990). These type of patients produce a smaller absolute number of errors and are more sensitive to the lexical status of the string (Arduino et al., 2002). Coherently, it has been concluded that a milder deficit accounts for the behavioral deficit expressed in substitution errors and that the two kinds of errors depend on a single mechanism, which can be disrupted along a continuum of severity (Mozer and Behrmann, 1990; Behrmann et al., 1991; Arduino et al., 2002).

However, Arduino et al. (2005), in describing RCG, a right-brain-damaged patient, who manifested a clear spatial reading disorder characterized mostly by left-sided substitutions without any other sign of USN, and in comparing the patient’s performance with other similar cases in the literature, suggested that substitution errors could not be directly related to unilateral spatial disorder. Moreover, he was sensitive to spacing, that is, by increasing the inter-letter space to three times the letter size, despite the fact that letters occupied a larger portion of the left neglected space, the total number of reading errors was halved. This finding suggested that substitution errors may depend at least in part on a specific mechanism and that perceptual integration may play a crucial role in the reading performance of brain-damaged patients. Accordingly, Martelli et al. (2011) proposed a dual model, stating that substitution and omission errors could be due to different mechanisms: the first is visuo-spatial in nature and is responsible for omissions in both ND and USN (such as errors in detecting left-sided elements in cancelation tasks); the second mechanism, which causes a predominance of substitutions, is perceptual and does not depend on neglect. In the latter case, substitution errors depend on a well-described feature integration mechanism that impairs recognition for above acuity letters moving toward the visual periphery and limits letter identification when other letters surround the signal (the so called *crowding* phenomenon). This phenomenon characterizes the normal periphery and amblyopic fovea (Irvine, 1945; Stuart and Burian, 1962) and psychophysical studies indicate that correct letter recognition can be restored by increasing letter spacing (for reviews, see Pelli et al., 2004; Whitney and Levi, 2011). Evaluating ND patients, Martelli et al. (2011) found that increasing letter spacing reduced substitution errors while increasing omissions. In line with the assumption that omissions are affected by a visuo-spatial deficit and substitutions by a perceptual one, the Authors also found that omissions, but not substitutions tended to be related to the severity of neglect, measured by several visuo-spatial tasks. By adopting Martelli et al.’s (2011) dual model it still remains to be explained what causes the occurrence of reading errors only in a fraction of patients with USN. In a recent study by Primativo et al. (2013) eye movements were recorded in neglect patients with and without ND and in a matched group of right-brain-damaged patients without neglect, while reading pseudowords and during a saccadic task with non-orthographic material. The results indicated that only ND patients (all characterized by left lateralized omission errors) showed a distorted eye movement pattern in both the reading task and the non-verbal saccadic task. The main feature of the abnormal oculo-motor pattern was characterized by a large amount of inaccurate fixations (i.e., more than 50% of ND patients’ fixations did not fall on the stimulus but they were distributed in different positions on the screen, both in the left and right hemispaces). The Authors also showed that USN patients without ND forced to read single words without eye movements produced a similar pattern of errors to that of ND patients with unlimited exposure time (i.e., left-sided errors). Primativo et al. (2013) concluded that the reading disorder in ND is the phenotypic expression of the exploratory deficit in USN when the fine eye movements required for reading are altered.

Accordingly, the two different error types would require specific diagnosis and treatments and a consequence of this hypothesis is that specific training for omission-type ND would aim to restore oculo-motor scanning, but would not improve reading in substitution-type ND.

Among all the possible techniques, we decided to adopt OKS (Pizzamiglio et al., 1990) since it facilitates the displacement of the oculo-motor exploration toward the neglected side of space and has the advantage of bottom-up techniques requiring neither consciousness of the deficit nor a goal-based behavior by the patient. This choice is also supported by recent studies which have shown that OKS significantly modulates many facets of the neglect syndrome, including ND, auditory neglect, subjective body midline, line bisection, and size distortions (Kerkhoff and Schenk, 2012) even though there are results which are not in accordance with such assumption (e.g., Antonucci et al., 1992; Pizzamiglio et al., 2004; Kerkhoff et al., 2006; Thimm et al., 2009).

In the present study two right-brain-damaged patients, with USN and no visual field defect, one affected by omission-type ND and the other affected by substitution-type ND, were selected by means of a pseudowords reading test (Vallar et al., 1996) and further investigated.

In order to confirm the relationship between omission errors and oculo-motor impairment, eye movements were recorded during a saccadic non-verbal task. Finally, the two patients were presented with a reading task before and after OKS (leftward moving dots) to test the sensitivity and specificity of the two types of reading errors to OKS.

### Case reports

**Figure 1:**
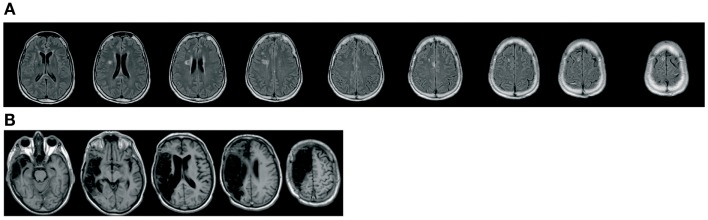
**MA (A) and EP (B) neuroradiological images**. The first patient shows a cortico-subcortical frontal lesion, while the latter has a huge fronto-temporo-parietal cortico-subcortical lesion.

MA, a 62-year-old female, right-handed, with 11 years of education. In October 2012, she suffered a subarachnoid hemorrhage from a ruptured aneurysm of the right internal carotid artery, preceded by an episode of loss of consciousness. She underwent endovascular embolization treatment. The TC scan revealed the presence of hypodensity at the level of both the right frontal cortex and periventricular white matter (insula, supplementary motor area, middle cingulum, superior frontal gyrus, inferior frontal operculum, rolandic operculum, putamen). No occipital damage was present (see Figure 1) and no visual field defect was present. At the first neuropsychological assessment, the patient appeared alert, well oriented, with some short-term memory difficulties, a tendency to confabulation, and a gaze deviation toward the right. She showed a moderate to severe USN. The speech was fluid and informative, abundant, and no aphasic disorders were detected. MA’s language comprehension was adequate for the demands of the present study. The performance in the naming tasks was not adequate, but was probably influenced by her visuo-spatial disorder. The speed of the lexicon access was reduced but within the limits. Performance in praxic-constructive tasks was insufficient, but again affected by the presence of neglect and perseverations. No evidence of visuo-perceptual integration deficit was observed (see Table 1 for demographic and the neuropsychological assessment information). Finally, she had a pathological performance at a words and pseudowords reading test (Vallar et al., 1996), characterized by omission errors (see Table 2).

**Table 1:** **Demographic features and baseline assessment for unilateral spatial neglect**.

Pat.	S	A	E	L	Letter Cancell.		Star Cancell.		Wundt-Jastrow		Sentence reading	Bisection
					Left	Right	Left	Right	Left	Right	
MA	F	62	8	F	42/53*	21/53	8/27*	3/27	4/20*	2/20	6/6*	5.4*
EP	M	60	10	FTP	4/53	1/53	13/27*	5/27	16/20*	0/20	1/6*	10.3*

F, frontal lobe; P, parietal lobe; T, temporal lobe; S, sex; M/F, male/female; A, age; E, educational level; L, lesion location; Scores: (i) cancelation tasks: omission errors; (ii) Wundt-Jastrow area illusion test: “unexpected” responses; (iii) reading task: the number of sentences in which patients showed left-sided errors; 16 cm lines bisection error (mm). *Pathological score; L, left; R, right.

**Table 2:** **Neglect dyslexia assessment (Vallar et al., 1996) for MA and EP**.

	MA		EP	
	Words	Pseudowords	Words	Pseudowords
Errors	18/38 (47.4%)	25/38 (65.8%)	2/38 (5.3%)	25/38 (65.8%)
Neglect errors	17/18 (94.4%)	25/25 (100%)	2/2 (100%)	13/25 (52%)
Omissions	16/17 (94.1%)	22/25 (88%)	0/2 (0%)	3/13 (23.1%)
Substitutions	0/17 (0%)	1/25 (4%)	2/2 (100%)	10/13 (76.9%)

Absolute number and % of errors are reported for all types of items misread, neglect errors, omissions, and substitutions. Neglect errors refer to all misread items with left-sided errors, according to the Caramazza and Hillis (1990) criterion. Omissions refer to all neglect errors in which the produced item length was shorter than the target. Substitutions refer to all neglect errors in which the produced item had the same length as the target.

EP, a 60-year-old male, right-handed, with 13 years of education. He suffered a cerebrovascular ischemic stroke, confined to the right hemisphere. A MRI scan (see Figure 1) identified a right fronto-temporo-parietal lesion (heschl gyrus, rolandic operculum, superior fronto-occipital fasciculus, inferior frontal operculum, superior longitudinal fasciculus, superior temporal gyrus, external capsule, supramarginal gyrus, insula, superior corona radiata, putamen, middle temporal gyrus, superior temporal pole, inferior parietal gyrus). No occipital damage was present and no visual field deficit was detected.

The failure of an attempt at mechanical unblocking of a middle cerebral artery thrombosis, associated with an intraparenchymal hemorrhage in the caudal part of the right putamen, without involvement of the internal capsule, led to a decompressive right craniectomy.

After 6 months he was cooperative and oriented in time and space. He presented a complete left hemiparesis and the neuropsychological assessment still showed impulsiveness, distractibility, reduced cognitive flexibility and planning difficulties, as well as a medium to severe USN for extrapersonal and peripersonal space, and visuo-constructional and visual-spatial skills deficits (see Table 1 for demographic and the neuropsychological assessment information). Finally, he showed ND by means of a words and pseudowords reading test (Vallar et al., 1996), characterized by substitution errors (see Table 2).

## Experiment 1

### Neglect dyslexia assessment

#### Material and procedure

The first experiment aimed to describe the type of reading errors in the two patients, according to the letter position analysis used by Martelli et al. (2011). Pseudowords were created by interchanging the syllables of existing words (taken from Burani et al., 2002; http://www.istc.cnr.it/grouppage/lexvar) in random positions in order to preserve pronunciation and minimize word similarity. We generated a list of 40, 5-to-8-letter pseudowords (10 for each length). The stimuli were written in capital Courier New font, which is characterized by consistent letter spacing. Letter size was kept constant (40 pt) and subtended 1.0°. Patients were shown two squared dots vertically displaced 1.5° apart in the center of the screen. These fixation marks remained on the screen for the entire experimental session. Stimulus onset was triggered when the patient steadily fixated the central marks for at least 50 ms. Each stimulus was presented at the center of the screen between the fixation marks (i.e., the central letter of each stimulus was vertically aligned to the fixation marks) and remained on the screen until onset of the patient’s response. There was no time constraint for responding. Patients were asked to read aloud each stimulus as accurately as possible. Pseudowords appeared in a randomized order across participants. Responses were digitally recorded and errors were scored after listening to the recorded track later.

#### Results

We measured the letter omissions and substitutions errors for each stimulus. Following Martelli et al. (2011) we applied a letter-based approach treating each letter in the word independently. Caramazza and Hillis (1990) criterion is strict in that it considers that no processing occurs on the left-side of the string and the processing is completely spared on the right of the neglect point. This criterion excludes from the analysis substitution errors occurring on the right side of the stimulus and gives a less detailed description of performance. Therefore, we measured the omission and substitution errors over the entire stimulus, following a letter-based analysis (Figure 1). By comparing the two criteria it emerges that: (1) Caramazza’s criterion underestimates the total number of omission and substitutions [e.g., the word “vacanza” (holiday) read fanza results in one omission error in that the production is shorter than the target, while according to a letter-based analysis two omissions, the letters v and a, and one substitution, f instead c, would be counted]. (2) Several errors although located on the left-side of the string are considered by Caramazza’s criterion “visual” errors [e.g., the word “elefante” (elephant) read “etepante” would be considered a visual error since it preserved the identity of the first letter, while according to a letter positional analysis it would be counted as two left-side substitutions, “l” as “t” and “f” as “p”).

Eye movements recording ensured that the first fixation landed on the center of the string. According to perceptual crowding the identificability of the letters falling around fixation and the external letters that only have one flanker nearby, should be spared when letter size is above acuity, as in the present case. Letters in intermediate positions should be unrecognizable because of crowding (Martelli et al., 2011). Thus we applied a two Gaussian distributions model to the data with picks on the left and right side of the centrally fixated string. On the converse if errors distribution is solely determined by the left lateralized neglect deficit data should be best described as an exponential decay.

**Figure 2:**
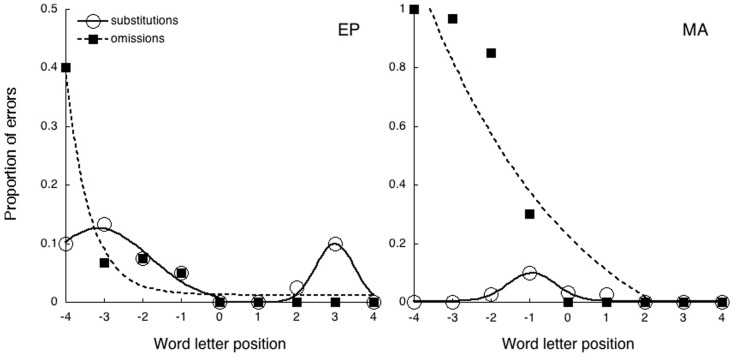
**The absolute number of reading errors made by the two patients, divided into substitutions and omissions as a function of letter position**.

Figure 2 reports the proportion of omission and substitution reading errors made by the patients as a function of letter position. From the figures it emerges that, while MA made a large number of omissions only on the left-side of the stimulus, EP made fewer errors, mostly substitutions, more evenly distributed across the entire stimulus. The same behavior has already been described in two other patients, AR and DNA (Martelli et al., 2011). The analysis of the error distribution in these two patients (Figure 2) showed that substitutions and omissions have different shapes as expected. The proportion of omission errors produced by MA and EP have been fit by a three parameters exponential decay model using the following equation

$$P_{o}=a+be^{\left(-c\;x\right)}$$

where *a* is the offset, *b* is the amplitude, and *c* is the rate of change.

In the case of EP the proportion of substitution errors has been fit by the sum of two Gaussian distributions according to the following equation

$$P_{s}=a+be\left(-{\left(x-c\right)}^{2}/d^{2}\right)$$

where *a* is the offset, *b* is the area under the curve, *c* is the center of the distribution, and *d* is the width. In the case of MA substitution errors a unimodal Gaussian distribution has been applied.

In the case of omissions the exponential decay captures a large proportion of variance for both observers (MA *R*^2^ = 0.94; EP *R*^2^ = 0.96). In this case errors are confined to the left-side of the stimulus as predicted by USN. Substitution errors show a substantially different pattern. In the case of EP the pattern is well captured by the bimodal distribution (*R*^2^ = 0.97) with picks at letter position –3.14 and 2.67, while the exponential fit doesn’t capture the shape of the distribution (*R*^2^ = 0.44). Errors are symmetrically distributed around the fixation point sparing the external letters that only have one flanker nearby (as predicted by crowding). In the case of MA the distribution is captured by a single Gaussian with a pick around letter position −1 (*R*^2^ = 0.88). These data are in agreement with previous findings by Martelli et al. (2011) in that patients characterized by a majority of substitutions generally produce fewer and distributed errors. Additionally, the data indicate that omissions but not substitutions show the clear left-lateralization typical of USN disorder.

## Experiment 2

### Eye movement in a non-verbal task

#### Material and procedure

As described in the introduction, Primativo et al. (2013) showed that the prevalence of omission errors in ND patients is associated with an impaired eye movement pattern. This was found not only during a reading task but also during a saccadic task which did not involve orthographic material but in which gaze simulated the sequential eye movements involved in reading. In order to assess whether a similar impairment is present in patient MA (who displays a prevalence of omissions) and thus could account for her reading difficulties, we conducted the same saccadic task used by Primativo et al. in which the patients had to follow a moving dot with their eyes on the horizontal meridian between five different spatial positions both right to left and left to right.

A black dot subtending 0.2° of visual angle and displayed on a white background, appeared along the horizontal meridian in five consecutive positions, 4.0° away from each other according to a synchronous paradigm (i.e., no gap). The dot appeared sequentially in the five positions and remained for 2 s in the two extreme positions and for 1 s in the three central ones. The sequence started with the extreme left dot and each dot appeared in turn until the extreme right dot appeared, then the reverse sequence took place. The rightward and leftward sequences were repeated twice in each trial. Three trials were administered. Patients were required to follow the dot as quickly and as accurately as possible.

Monocular eye movements were recorded in binocular vision via an SR Research Ltd., Eye Link 1000 eye tracker (SR Research Ltd., Mississauga, ON, Canada) sampling at 500 Hz, with spatial resolution of less than 0.04°.

Head movements were avoided by using a headrest.

Patients sat 57 cm away from a 17″ CRT monitor. A standard nine-point calibration procedure was run before collecting the data. The calibration targets were presented randomly in different positions on the screen. The experimental task started immediately after calibration.

Eye movement data were processed using EyeLink Data Viewer software (SR Research Ltd., Mississauga, ON, Canada).

#### Results

**Figure 3:**
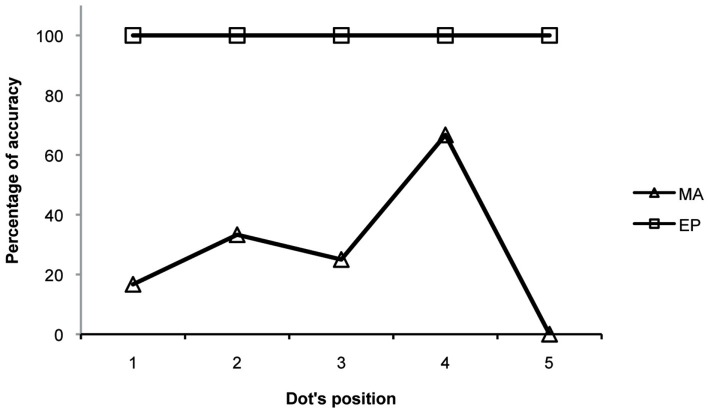
**The mean percentage accuracy in a non-verbal saccadic task (following a dot moving from left to right and from right to left) made by the two patients**.

Accuracy (mean percentage of fixations on the dot when it was on the screen, in both directions, Figure 3) was measured.

**Figure 4:**
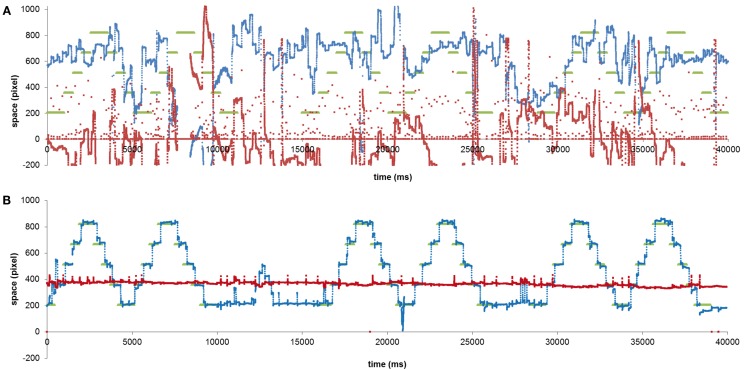
**MA (A) and EP (B) ocular behavior in a non-verbal saccadic task**. The green lines indicate the dot positions, the red lines indicate the *y* coordinate of the eye movements and the blue lines the *x* coordinate.

Figure 4 shows the ocular behaviour of MA and EP during the saccadic task. We excluded analysis of fixations made on the first dot in the sequence and analysis of anticipatory saccades (i.e., saccades starting before the appearance of the following dot). We also excluded analysis of fixations that were far from the target with respect to its vertical axis (i.e., over 2 SD calculated on the vertical fixation positions of a control group collected and described in Primativo et al., 2013). The remaining fixations were considered “accurate” if they fell no more than 1° of visual angle away from the target.

MA and EP data were compared to that of four right-brain-damaged patients, by means of Crawford statistics (Crawford et al., 1998; Crawford and Garthwaite, 2002). The control subjects, one female and three males, were comparable in terms of age (mean age = 68.5 years, range 52–78) and education (mean education = 11.8 years; range 8–18) to the patients. The analyses of accuracy (Table 3) revealed that MA was significantly less accurate than the controls both when the dot was moving rightward and leftward [left to right: *t*(3) = −55.365; *p* = 0.00001; right to left: *t*(3) = −15.426; *p* = 0.00059; all: *t*(3) = −28.235; *p* = 0.0001], while EP did not significantly differ from the controls [left to right: *t*(3) = 0.000; *p* = 1; right to left: *t*(3) = 0.446; *p* = 0.68573; all: *t*(3) = 0.447; *p* = 0.68504].

**Table 3:** **Comparisons between the accuracy (% correct) of each one of the two patients affected by USN and ND and four right-brain-damaged patients without USN and ND (controls), in the conditions where the dot moved from left to right, from right to left, and in the two conditions together**.

	DOT direction (% accuracy)		
	Left-right	Right-left	All
Controls	100.00	97.92	98.96
EP	100.00	100.00	100.00
MA	38.10**	26.00**	33.30**

*p < 0.05; **p < 0.01.

**Table 4:** **Comparisons between the accuracy (% correct) of each one of the two patients affected by USN and ND and four right-brain-damaged patients without USN and ND (controls), for each dot position**.

	DOT position (% accuracy)				
	1	2	3	4	5
Controls	95.83	97.92	100.00	100.00	100.00
EP	100.00	100.00	100.00	100.00	100.00
MA	16.70**	33.30**	25.00**	66.70*	0.00**

*p < 0.05; **p < 0.01.

The analyses of accuracy for the dot position (Table 4) revealed that MA was less accurate at each dot position [first: *t*(3) = −0.448; *p* = 0.68469; second: *t*(3) = −13.86; *p* = 0.00081; third: *t*(3) = −67.082; *p* = 0.00001; fourth: *t*(3) = −29.784; *p* = 0.00008; fifth: *t*(3) = −89.443; *p* = 0.00000], whereas none was different from the controls in the case of EP’s fixations [1°: *t*(3) = −8.497; *p* = 0.00034; 2°: *t*(3) = 0.446; *p* = 0.68573; 3°: *t*(3) = 0.000; *p* = 1; 4°: *t*(3) = 0.000; *p* = 1; 5°: *t*(3) = 0.000; *p* = 1].

MA was profoundly impaired in performing a simple saccadic task on the horizontal axis. Although this result might be interpreted as a sign of premotor neglect (e.g., Saevarsson, 2013), the result that MA’s performance was impaired in both directions (toward the ipsilesional side as much as toward the contralesional side) is unlikely to support this hypothesis. Moreover, the same result was obtained by Primativo et al. (2013), who showed how ND patients mainly characterized by letter omission errors showed both USN and an oculo-motor impairment.

On the other hand, EP, who was affected by USN and ND, as well, did show a preserved performance at the same saccadic task, confirming that substitution-type ND is a qualitatively different disorder to omission-type ND.

## Experiment 3

### Optokinetic stimulation effect

#### Material and procedure

The third experiment aimed to verify the effect of the OKS on ND and in particular to assess whether MA and EP, characterized by two different types of reading errors had a different sensitivity to it.

The OKS consisted of random black dots of 0.75° in diameter presented on a gray background of 16° cd/m^2^ in luminance, moving from right to left with a speed of 11.3°/s. Before and after the OKS, two sets of 30 pseudowords of different length (6-7-8 letters; font: Courier New; font size: 22) were presented at the center of a CRT 17″ monitor screen (1024 × 768 pixel), without a fixation point.

Two lists of pseudowords were used in order to avoid repetition and learning effects. The lists were constructed so as to preserve pronunciation and minimize word similarity (as in experiment 1). The two lists were matched for all relevant psycholinguistic variables such as length in terms of number of letters and syllables, bigram frequency, neighborhood size, and first phonemes and contained different stimuli from those of experiment 1.

The patients were seated in a dark and silent room facing a monitor displaying centrally presented visual stimuli. Their heads were positioned in an adjustable head-and-chin rest so that the distance between their eyes and the screen was approximately 57 cm. The experiment and the recording of the responses were carried out with MatLab 7.13.

The experimental session consisted of a reading task, before and after OKS. In each condition the patients had to read aloud 30 pseudowords presented at the center of the screen, written in white on a gray background. No fixation point was used. There were no time constraints and the 30 pseudowords were presented in the same fixed sequence for both patients. Only reading errors were recorded.

The same reading task was also presented to a control group of 10 healthy individuals who made no errors.

The experimental procedure consisted of two parts: a pseudowords reading task before the OKS (a), 10 min of OKS (b) and a pseudowords reading task (with different pseudowords) after the OKS (c).

During the OKS (b) the patients’ task was to look at the screen with the moving dots, with the instruction not to fixate on any specific dot.

#### Results

Given that the performance of healthy subjects represented a ceiling in the pseudowords reading task, the chi-square analysis was used to test whether the number of reading errors was significantly different between the experimental conditions (before and after the OKS) in each patient and for each type of error.

In the pre-OKS condition MA misread 19 out of 30 pseudowords. According to a letter-based analysis she omitted 25 letters in 19 pseudowords. In the post-OKS condition MA misread 12 out of 30 pseudowords. In this condition she omitted 12 letters in the 12 misread pseudowords [a reduction of omission errors from 63.3 to 40%, χ^2^(1) = 5.136; *p* = 0.023].

MA showed a significant reduction in the number of omitted letters in the post-OKS stimulation compared to the pre-OKS condition [χ^2^(1) = 6.72; *p* = 0.0095], while substitutions (pre-OKS: 1; post-OKS: 0) were at ceiling level. Conversely, EP did not show any significant difference in terms of the number of substituted letters [χ^2^(1) = 0.08; *p* = 0.7728] or omitted letters [χ^2^(1) = 0.25; *p* = 0.617].

He misread 14 out of 30 pseudowords in the pre-OKS condition, making 13 substitutions in 12 pseudowords and 5 omissions in 5 pseudowords. In the post-OKS condition EP misread 10 out of 30 pseudowords. According to a letter-based analysis, he substituted 11 letters in 10 pseudowords and omitted 3 letters in 3 pseudowords [a reduction of omission errors from 16.7 to 10%, χ^2^(1) = 1.816; *p* = 0.178, and a reduction of substitution errors from 40 to 33.3%, χ^2^(1) = 0.671; *p* = 0.413].

Both letter and word based analyses showed a significant reduction only in the case of MA omission errors.

**Figure 5:**
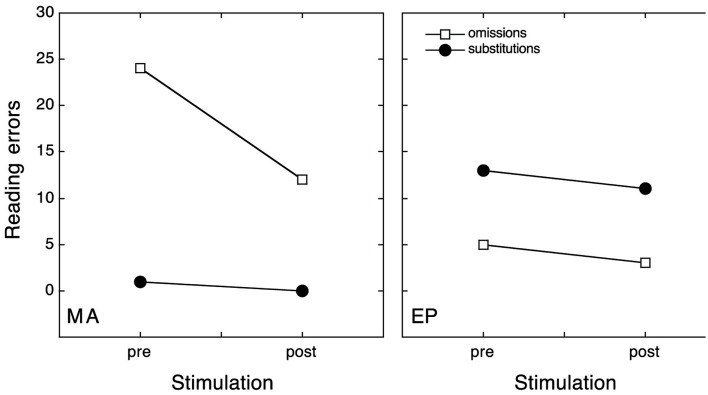
**The absolute number of letters omitted (open square) or substituted (filled dots) on the left-side of the stimulus while reading pseudowords, before and after OKS**.

These results confirm the hypothesis of a dissociation in terms of the sensitivity to stimulation between the two types of reading errors, such that only omissions-type ND was affected by the OKS (see Figure 5).

## Discussion

Two patients affected by USN and ND were evaluated with a version of OKS (Pizzamiglio et al., 1990) presented with a small display (Reinhart et al., 2011) in order to validate the hypothesis that omissions could benefit from the slow leftward movement induced by this kind of stimulation. The two patients were identified as having ND using a words and non-words reading task (Vallar et al., 1996) and were then given a pseudowords reading task (experiment 1), and a non-verbal saccadic task (experiment 2) to assess the distribution of errors and their oculo-motor behavior.

One of the two patients, MA, showed mainly omission errors, an exponential distribution toward the contralesional side of space and an oculo-motor impairment at the non-verbal task in both spatial directions (as were all six ND patients described by Primativo et al., 2013). On the other hand, EP, did not show any exploratory deficit and his ND was mostly characterized by substitution errors, distributed in a bimodal manner (as were AR and DNA patients described by Martelli et al., 2011).

The result that substitution-type ND, in contrast to omission-type ND, was not associated with oculo-motor impairment, represents a new result and, even though it needs further evidence by group studies, it supports Martelli and Collaborators’ dual model of ND.

As expected, only MA was shown to be sensitive to OKS and after 10 min of leftward moving dots stimulation, showed a significant reduction in omission errors, both at letter and word level.

Reinhart et al. (2011) found a similar result with paragraph reading. Leftward OKS was effective in reducing word omission errors, but not stimulus-centered errors. Their distinction is theoretically made on the basis of the model by Caramazza and Hillis (1990) and, from a phenomenological point of view their stimulus-centered errors included both omission and substitution errors on single words reading while omission errors alluded to the omission of entire words when reading texts.

This result suggests a double dissociation between word and sentence reading which is still a matter of debate (Vallar et al., 2010; Friedmann et al., 2011). However, the data are not helpful in assessing the specific effect of OKS since the authors did not distinguish between letter error types.

They conclude that OKS effectiveness on word omissions is due to a triggering of (pre-)attentional processes toward the contralesional side of egocentric space. Nevertheless this account is not specific to ND and could be the reason why it has been shown to be effective also with other USN symptoms of visual and auditory neglect (Antonucci et al., 1992; Pizzamiglio et al., 1996; Kerkhoff et al., 2006; Thimm et al., 2009).

Here we suggest that a more specific mechanism is involved in ND. In the light of Primativo et al.’s (2013) results, letter omissions are due to the co-occurrence of USN and altered oculo-motor exploration, so, the automatic pursuit eye movements associated to OKS, could act specifically and directly to compensate or restore that mechanism. Indeed, our results show that OKS was able to benefit the specific exploratory behavior of the patient with ND characterized by omissions, by helping her in a single item reading task.

According to our hypothesis, OKS should be effective only for omissions but not for substitution errors.

Pizzamiglio et al. (2004) found a positive effect of OKS only on individual patients and the authors tried to determine if some characteristics could be linked to the effectiveness of OKS.

They considered the Barthel Index, visual field defect and motor impairment but none of those predictive variables could discriminate significantly between patients experiencing an improvement with OKS and patients showing no benefit. Unfortunately, they did not consider specific deficits of USN such as ND. An alternative interpretation of these results could be found in a model that was proposed by Ellis et al. (1993), which argued that omissions could reflect the co-presence of left ND and left homonymous hemianopia, whereas substitutions could reflect the pure presence of left ND without hemianopia. In the second case, residual information may activate contralesional positional coding of graphemes at the graphemic level. However, since it is true that many cases are in accordance with these predictions, other more recent studies have shown that this is not always the case (e.g., patient SVE by Miceli and Capasso, 2001; Martelli et al., 2011). In particular, in both Martelli et al. (2011) and in the present study, the absence of hemianopia was the condition *sine qua non* to participate in the research. The reason for this choice was precisely to avoid such a confounding variable.

In particular, it is also evident that MA (the patient with an omission-type ND) has a very small and anterior brain lesion, not compatible with a visual field defect.

Our study cannot shed light on the anatomical location for the two types of ND errors given that we had just two patients and they presented two very different lesions in terms of extension. Both of them showed cortical and subcortical frontal lesions, but EP showed a much bigger fronto-parieto-temporal lesion. In particular, the lesions of the two patients overlap on insula, putamen, inferior frontal operculum, and rolandic operculum, while they do not share the involvement of superior frontal gyrus, supplementary motor area, and middle cingulum (MA), other than the parieto-temporal areas (EP).

While further research will help in addressing the anatomical correlates issue, we think that our study suggests an interesting new approach to the treatment of reading errors in neglect patients.

Indeed, in contrast to the usual approach to USN rehabilitation, which considers the deficit to be due to the same core mechanism, we propose an approach to the rehabilitation of neglect in which symptoms and specific mechanisms are treated in a specific way.

Our study was not designed to be a full rehabilitation program, since this would require different methodologies and almost 10 sessions of OKS. Our aim was different: we wished to verify the sensitivity to OKS of different types of single stimuli reading disorder associated with USN.

In particular, here we presented a dissociation in the transient effects of OKS between omission and substitution types of ND. A systematic procedure is needed in the short to test its effectiveness in the rehabilitation of ND patients and the presence of long lasting effects.

## Conflict of Interest Statement

The authors declare that the research was conducted in the absence of any commercial or financial relationships that could be construed as a potential conflict of interest.
